# Biomechanical Analysis and Mid-Term Clinical Outcomes of the Dynamic-Transitional Optima Hybrid Lumbar Device

**DOI:** 10.3390/jcm14228087

**Published:** 2025-11-14

**Authors:** Shih-Hao Chen, Shang-Chih Lin, Chi-Ruei Li, Zheng-Cheng Zhong, Chih-Ming Kao, Mao-Shih Lin, Hsi-Kai Tsou

**Affiliations:** 1 Department of Orthopedics, Dalin Tzu Chi Hospital, Buddhist Tzu Chi Medical Foundation, Chiayi 622, Taiwan; shihhaotzuchi@gmail.com; 2 Graduate Institute of Biomedical Engineering, National Taiwan University of Science and Technology, Taipei 106, Taiwan; 3 Department of Neurosurgery, Neurological Institute, Taichung Veterans General Hospital, Taichung 407, Taiwan; 4 Department of Mechanical Engineering, National Chiao Tung University, Hsinchu 300, Taiwan; 5 Department of Orthopedics, Kaohsiung Municipal Ta-Tung Hospital, Kaohsiung Medical University, Kaohsiung 801, Taiwan; 6 Functional Neurosurgery Division, Neurological Institute, Taichung Veterans General Hospital, Taichung 407, Taiwan; 7 Department of Post-Baccalaureate Medicine, College of Medicine, National Chung Hsing University, Taichung 402, Taiwan

**Keywords:** dynamic stabilization, dynamic-transitional optima, transition segment, finite element analysis, minimally invasive surgery, facet contact force, disc degeneration, interpedicular travel, hybrid spinal instrumentation

## Abstract

**Background/Objectives**: Spinal fusion with static fixation—surgically joining two or more vertebrae to eliminate motion—is commonly employed to treat degenerative spinal disease. However, the rigidity imposed by static constructs and the increased load on the adjacent segments frequently result in complications such as disc or facet degeneration, spinal stenosis (SS), and segmental instability. This study investigates the effectiveness of pedicle-based dynamic stabilization using the Dynesys system, particularly in a dynamic-transitional optima (DTO) hybrid configuration, in mitigating adjacent segment disease (ASD) and improving clinical outcomes. In this work, we analyzed the mechanical performance and intermediate-term clinical effects of the DTO hybrid lumbar device, focusing on how the load-sharing properties of the Dynesys cord–spacer stabilizers may contribute to junctional complications in individuals with diverse grades of intervertebral disc degeneration. Study Design/Setting: We designed a combined biomechanical finite element (FE) and experimental analysis to predict the clinical outcomes. Patient Sample: Among 115 patients with lumbar SS enrolled for analysis, 31 patients (mean age: 68.5 ± 7.5 years), with or without grade I spondylolisthesis (18/13), underwent a two-level DTO hybrid procedure—L4–L5 static fixation and L3–L4 dynamic stabilization—with minimal decompression to preserve the posterior tension band. Post-surgical follow-ups were conducted for over 48 months (range: 49–82). Outcome Measures: Radiological assessments were performed by two neurosurgeons, one orthopedic surgeon, and one neuroradiologist. The posterior disc height, listhesis distance, and dynamic angular changes were measured pre- and postoperatively to evaluate ASD progression. **Methods**: Dynamic instrumentation was assigned to the L3–L4 motion segment with lesser disc deterioration, in contrast to the L4–L5 segment, where static fixation was applied due to its greater degree of degeneration. FE analysis was performed under displacement-controlled conditions. Intersegmental motion analysis was conducted under load-controlled conditions in a synthetic model. **Results**: The DTO hybrid devices reduced stress and motion at the transition segment. However, compensatory biomechanical effects were more pronounced at the adjacent cephalad than the caudal segments. In the biomechanical trade-off zone—where balance between motion preservation and stabilization is critical—the flexible Dynesys cord significantly mitigated stiffness-related issues during flexion. At the L3–L4 transition level, the cord–spacer configuration enhanced dynamic function, increasing motion by 2.7% (rotation) and 12.7% (flexion), reducing disc stress by 4.1% (flexion) and 12.9% (extension), and decreasing the facet contact forces by 4.9% (rotation) and 15.6% (extension). The optimal cord stiffness (50–200 N/mm) aligned with the demands of mild disc degeneration, whereas stiffer cords were more effective for segments with higher degeneration. The pedicle screw motion in dynamic Dynesys systems—primarily caused by axial translation rather than vertical displacement—contributed to screw–vertebra interface stress, influenced by the underlying disc or bone degeneration. **Conclusions**: Modulating the cord pretension in DTO instrumentation effectively lessened the interface stress occurring at the screw–vertebra junction and adjacent facet joints, contributing to a reduced incidence of pedicle screw loosening, ASD, and revision rates. The modified DTO system, incorporating minimal decompression and preserving the posterior complex at the dynamic level, may be biomechanically favourable and clinically effective for managing transitional degeneration over the mid-term.

## 1. Introduction

Spinal fusion using static fixation is frequently performed to address lumbosacral instability, particularly when caused by degenerative conditions. However, successful radiographic fusion does not always correlate with clinical improvement. This disconnect often results from degenerative changes in the adjacent mobile segments due to increased mechanical stress and altered kinematics. Such adjacent segment pathology may manifest as disc or facet joint degeneration, spinal stenosis (SS), ligamentous laxity, or construct instability. The inherent rigidity of pedicle-based static fixation systems significantly contributes to junctional problems, leading to revision surgery rates exceeding 30% within 10 years—a consequence of constrained segmental motion and compensatory hypermobility at adjacent levels [1,2,3,4].

The Dynesys system (Zimmer Inc., Warsaw, IN, USA) is a pedicle-based dynamic stabilization (PDS) implant designed to maintain segmental motion while providing internal bracing. It employs high-polymer spacers and tensioned cords to create specific screw–spacer and spacer–cord linkages, restoring near-physiological kinematics at pathological segments [5,6,7,8,9,10]. Building on this technology, the dynamic-transitional optima (DTO) hybrid system combines static fixation at unstable levels with dynamic Dynesys stabilization at adjacent transitional segments. This hybrid approach alleviates adverse adjacent-level effects and prevents iatrogenic degeneration associated with rigid constructs [11,12,13,14,15,16,17].

Biomechanically, PDS systems such as Dynesys enhance stability during flexion, extension, and lateral bending while restricting axial rotation [18]. The dynamic component of the DTO system features a flexible spring cord pretensioned with a size-matched spacer to secure the screw–spacer interface. This configuration is intended to preserve mobility and distribute the load-bearing forces more evenly, reducing the stress on intervertebral discs and facet joints. However, four major issues require further investigation in evaluating hybrid constructs: the effectiveness of hybrid devices in mitigating junctional problems; the influence of the severity of disc and facet degeneration on hybrid device selection; the impact of screw–vertebra interfacial stress on fatigue, particularly in osteoporotic bone; and the optimization of surgical techniques to enhance the long-term functional outcomes.

Despite the increasing use of DTO systems, no comprehensive study has combined both biomechanical and clinical evaluations, particularly with respect to the transitional and adjacent segments in various disc/facet degeneration. With this study, we aim to fill this gap by examining the biomechanical performance of DTO constructs under various cord pretension configurations to identify a biomechanical “trade-off” region—the range where motion preservation is balanced with stabilization to minimize junctional stress [19]. Finite element (FE) modelling and experimental load-controlled tests are performed to analyze intersegmental motion under longitudinal load transfer [20]. In addition, a literature review of eight clinical studies on DTO applications was conducted to assess the indications, rates of screw loosening, adjacent segment disease (ASD), and surgical revision rates. We also propose a modified surgical technique utilizing minimally invasive surgery (MIS) combined with posterior complex preservation at the dynamically stabilized level. This approach is intended to reduce the implant–bone interfacial stress and lower the ASD incidence across different stages of degeneration [21]. Our findings can be used to clarify factors influencing the biomechanical and clinical performance of DTO constructs, offering guidance toward achieving optimal patient outcomes.

## 2. Materials and Methods

### 2.1. Finite Element (FE) Simulation of Degenerative Lumbosacral Spine Under Loading

**Figure 1:**
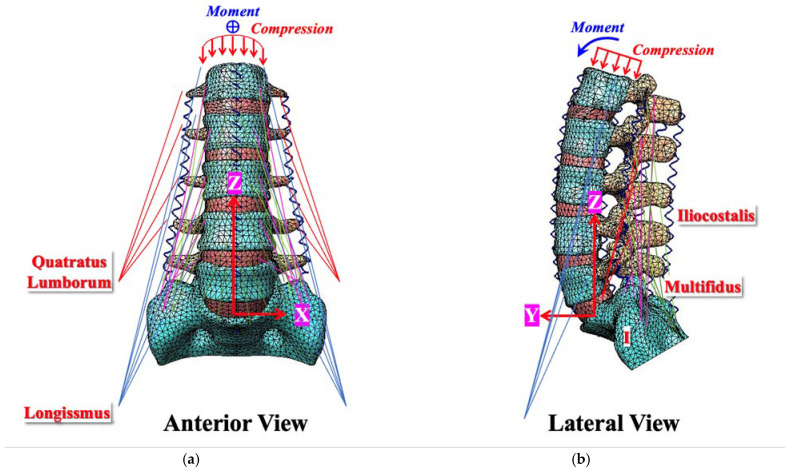
Simulation of lumbar motion using a displacement-controlled finite element model. A lumbosacral finite element model was used to simulate four types of lumbar motion (flexion, extension, lateral bending, and rotation) under a “displacement-controlled” mode. This mode was applied at the top of the L1 vertebra using the compression, moment, and contracture of five muscle groups, including the quadratus lumborum, iliopsoas, longissimus, iliocostalis, and multifidus. (**a**) Anterior view. (**b**) Side view.

**Figure 2:**
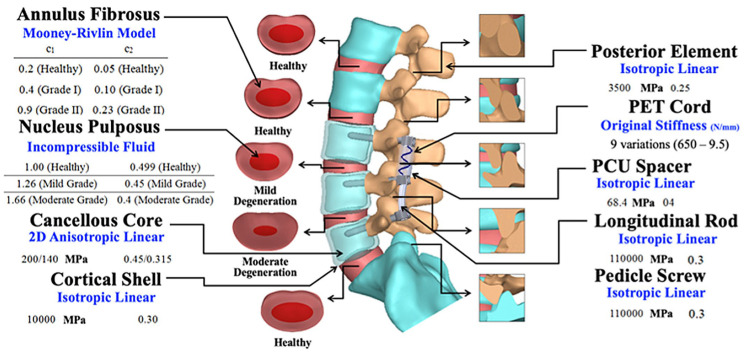
Anatomical and material composition of the lumbosacral finite element model. The lumbosacral finite element model included 43 components (bones, discs, and implants), 46 muscles, and 45 ligaments. Young’s modulus and Poisson’s ratio were applied as previously described [19]. Disc–facet joint degeneration was simulated within this musculoligamentous model to more closely represent clinical conditions.

A lumbosacral FE simulation was developed and calibrated according to cadaveric data and numerical simulations based on previously established intact and degenerative spine models [19]. The intervertebral disc was modelled to include the annulus fibrosus, represented as a Mooney–Rivlin hyperelastic material, and the nucleus pulposus, defined as an incompressible fluid-filled cavity. These structures were enclosed by the endplates of the adjacent vertebrae, each with a thickness of 1 mm. To simulate healthy facet joints, paired articulating surfaces were prepared with a precise 0.5 mm gap, allowing for separation and frictionless sliding while imparting only orthogonal forces during motion [22]. Moderate degeneration at the L4–L5 disc–facet joint was modelled with a 33% reduction in the disc height, a 40% increase in the annulus area, a 66% increase in the nucleus modulus, and a 0.3 mm decrease in the facet gap. Mild degeneration at L3–L4 was represented by a 15% reduction in the disc height, a 16% increase in the annulus area, a 26% increase in the nucleus modulus, and a 0.4 mm decrease in the facet gap [23]. Tension-only elements with strain-dependent springs simulated the ligamentous structures, including the supraspinous and interspinous ligaments, ligamentum flavum, and articular facet capsules, linking the adjacent vertebrae. Local musculature, excluding the abdominal muscles, was modelled based on previous studies [24,25], including the spatial structures of the quadratus lumborum, iliopsoas, longissimus, iliocostalis, and multifidus (Figure 1). A compressive load of 500 N together with moments of 10 Nm in flexion, extension, lateral bending, and axial rotation were applied at the superior surface of the lumbosacral spine. A Cartesian coordinate system (*x-y-z*) was set at the bottom centroid of the L5 vertebra to describe six-degrees-of-freedom motion [19,26]. In total, 45 ligaments and 46 muscles were symmetrically simulated along the sagittal plane (Figure 2), enabling the comprehensive evaluation of the DTO fixation performance under load with varying degeneration conditions.

### 2.2. DTO Fixation Setup in FE and Experimental Models Using Indices for Analysis

A Dynesys cord, pretensioned to 300 N, was integrated through a cannulated spacer (20 mm in length, 11 mm in outer diameter, 2 mm in thickness) to ensure a secure fit at the screw–spacer junction of the L3–L4 transition segment. Additionally, a preset 15° lordotic rod was statically secured at the L4–L5 segment. By varying the spring cord stiffness from its baseline value of 650 N/mm, the trade-off behaviour linked to junctional complications was examined [19,26]. The fixator components were defined as exhibiting linear elastic and isotropic behaviour. Local mesh refinement was applied at the fixation interfaces and other highly stressed regions to evaluate the von Mises stresses against their respective yield strengths. To prevent abrupt failure and unrealistically high stress concentrations, the element quality was monitored using aspect ratio and Jacobian checks, ensuring robust monotonic convergence. Simulation Ed. 2011 software (SolidWorks Corporation, Concord, MA, USA) was used to perform analyses with a nonlinear algorithm that included a large-deformation formulation and a direct sparse solver. The intact degenerative, static, and hybrid models comprised about 10,300, 13,000, and 20,000 elements, respectively.

**Figure 3:**
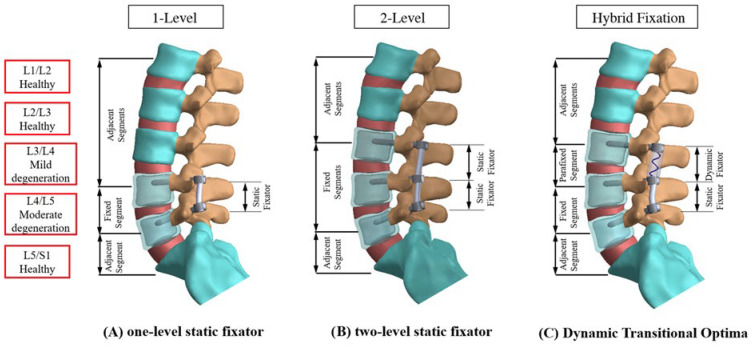
Comparison of fixation strategies at L3–L5 in finite element simulations. For biomechanical comparison, three models were simulated using different configurations of static and dynamic fixation across the L3–L5 segments: (**A**) one-level static fixation, (**B**) two-level static fixation, and (**C**) two-level dynamic-transitional optima fixation.

The applied loads were iteratively adjusted to maintain a consistent total disc range of motion (ROM)—achieved via a follower preload—across intact degenerative models, allowing comparison between one-level, two-level static, and hybrid devices [20,25,26,27] (Figure 3). A displacement-controlled approach was adopted, and the computed responses were normalized to those of the corresponding degenerative L3–L4 and L4–L5 models without instrumentation. To evaluate the hybrid behaviour, four key metrics were analyzed: disc ROM (represented by Euler angle variations under each loading condition), disc stress (mean stress values across the annulus and nucleus), facet contact force (FCF), and stress pattern along the bone–screw interface, with special attention to the transition segment. Additionally, the load transmission across the cord–spacer and vertebrae were numerically analyzed by evaluating three-dimensional interpedicular travel (IPT) and interpedicular displacement (ID) to confirm the device’s sensitivity to disc degeneration [28]. The potential risk of screw loosening was also inferred from the contact shear stress along the pedicle screw’s trajectory during motion.

### 2.3. Intersegment Kinematics Survey of DTO in Distinct Scenarios

**Figure 4:**
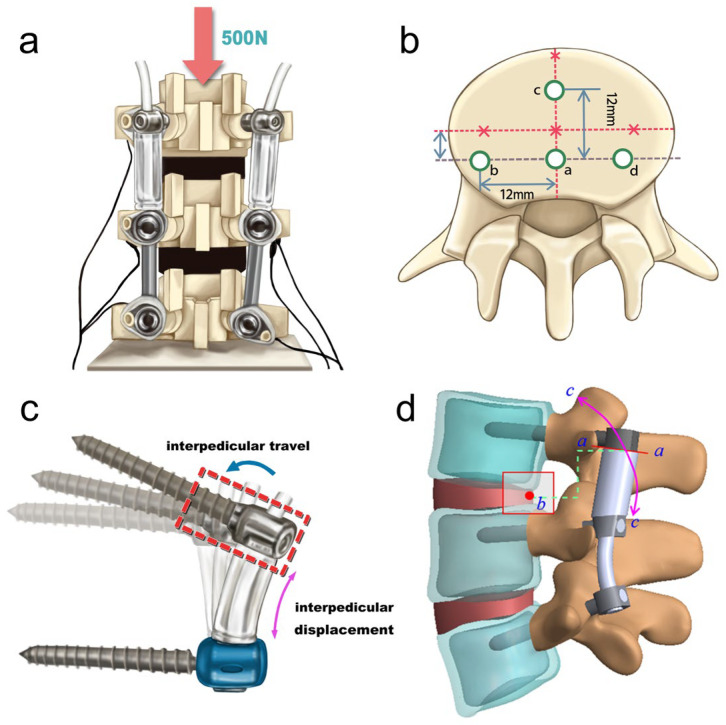
Experimental setup for evaluating intersegmental motion and device stress. (**a**) Intersegmental motion behavior was experimentally analyzed on a synthetic model under a “load-controlled” mode. Infrared light-emitting diodes were bilaterally mounted on each verte-bral body to record three-dimensional coordinates in the dynamic-transitional optima (DTO) de-vice. Initial 2 mm precompression ensured construct stability and increased axial vertical load to 500 N. (**b**) A 10 Nm bending moment—either in flexion (ac) or lateral bending (ab and ad)—was then applied to (**c**) measure interpedicular travel vector and interpedicular displacement. (**d**) Under 300 N of cord pretension, the screw–spacer joint (green) followed a motion curve (purple, cc) around the center of rotation (red dot, b) that allowed for tracking the contact stress area (red, aa) on the spacer tube.

A synthetic model was established to examine the interpedicular motion of the DTO fixator’s dynamic module, following a modified ISO 12189 testing protocol [29], incorporating custom-made discs to provide anterior support for the vertebral testing blocks. The intervertebral discs had stiffness values of 600 or 1100 N/mm at the transition segment to simulate varying grades of disc degeneration, while a fixed stiffness of 1600 N/mm was introduced at the fixed segment to evaluate the two-level hybrid construct [20]. A preliminary 2 mm precompression of the assembled construct ensured device stability, thereby increasing the axial vertical load to 500 N. Each vertebra was bilaterally equipped with infrared light-emitting diodes to track the three-dimensional coordinates of the DTO kinematic performance using a motion tracking apparatus (Phoenix Technology, Vancouver, BC, Canada) (Figure 4a,b). A load-controlled protocol was employed by applying a 10 Nm bending moment in flexion or lateral bending, repeated across five iterations. Average values were obtained to quantify the IPT and ID (Figure 4c). The spatial modelling of the IPT vector *r* (*r* =$\sqrt{r_{x}^{2}+r_{y}^{2}+r_{z}^{2}}$) in Cartesian coordinates (*x*, *y*, *z*) was used to evaluate the three-dimensional interpedicular screw movement at the transition segment. The ID was defined as the longitudinal migration of the flexible cord/spacer, indicating the axial stiffness of the DTO device under constraint. Kinematic patterns were analyzed by assessing the dimensional differences in IPT and ID magnitudes across different disc degeneration scenarios. A paired *t*-test was conducted to determine statistical significance, with a *p*-value < 0.05 considered significant.

### 2.4. Patient Selection and Clinical Analysis of Two-Level Hybrid Instrumentation

A total of 115 patients with lumbar SS were enrolled between 2012 and 2018 after obtaining authorization from the Institutional Review Board (IRB No. CE22412B). Patients were excluded if they had multilevel (more than one segment fusion or dynamization) hybrid instrumentation (n = 35), were under 60 years of age (n = 23), had disruption of spinal process ligaments at the transition level (n = 21), presented with infection (n = 2), or were lost to follow-up (n = 3). Ultimately, 31 patients (mean age = 68.5 ± 7.5 years), with (n = 18) or without (n = 13) grade I spondylolisthesis, underwent two-level hybrid DTO surgery. This consisted of static fixation at L4–L5 and dynamic fixation at L3–L4, with the dynamic cord pretensioned to 200–300 N based on the degree of disc degeneration. All patients underwent minimal decompression (of the L3–L4 stenosis) to preserve the tension band mechanism and were followed for over 48 months (range: 49–82) postoperatively. The novel MIS procedure included bilateral L3–L5 laminotomy, preservation of the posterior ligament complex, and L4–L5 transforaminal interbody fusion through a posterior midline incision under general anesthesia. Decompression was achieved while maintaining the lumbar spine’s neutral alignment. To prevent instability at the dynamic L3–L4 level, the spinous process, supraspinous ligament, and at least two-thirds of the facet joints were carefully preserved. Radiological assessments were independently conducted by two neurosurgeons, one orthopedic surgeon, and one neuroradiologist. These evaluations included measurements of the posterior disc height, spondylolisthesis distance, and angular motion on dynamic flexion–extension lateral radiographs at the L2–L3, L3–L4, and L5–S1 levels. Imaging was performed immediately post operation and at 12, 24, and 48 months after surgery. Radiographic signs of failure, including screw loosening/breakage, adjacent segment pathology, or the need for revision surgery, were also documented.

## 3. Results

### 3.1. Evaluation of Junctional Pathologies in Posterior Lumbar Fixation Systems

**Figure 5:**
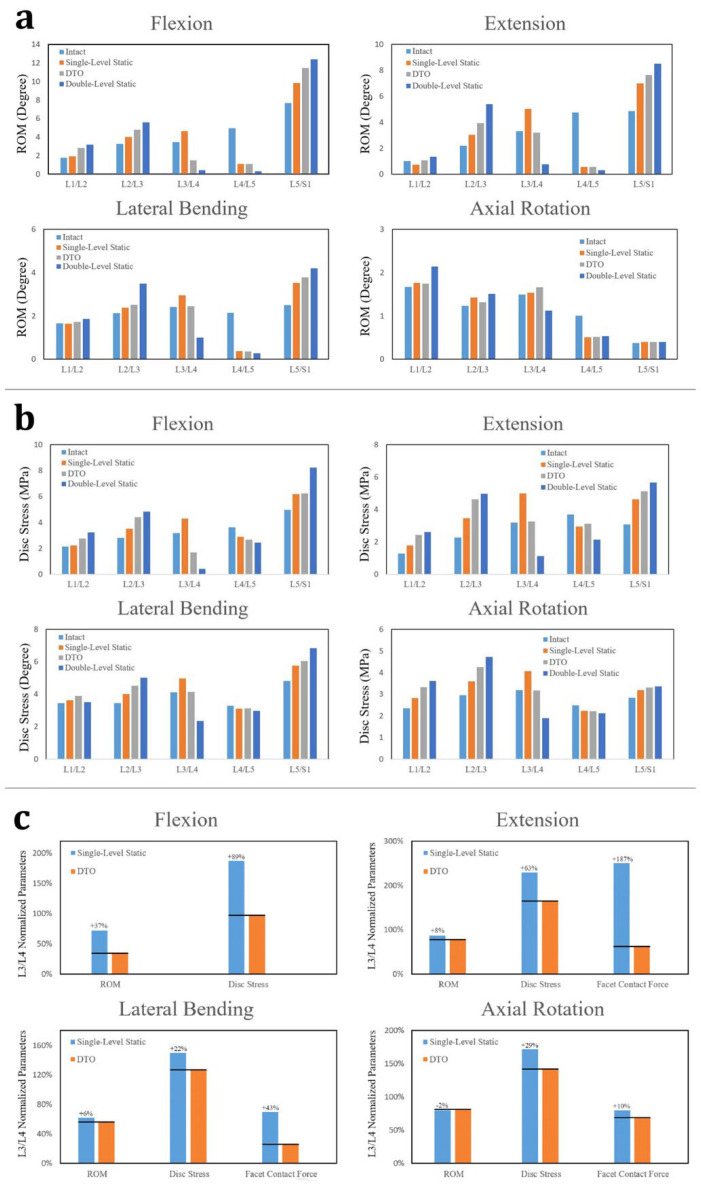
Comparative analysis of the fixation strategies on the junctional biomechanics. The DTO, one-level, and two-level rigid fixation constructs demonstrated different severities of junctional complications, more frequently at the superior level (L2–L3) than at the inferior level (L5–S1). The DTO dynamic mixed-design fixator outperformed the two-level static fixator, as shown by the following: (**a**) the ROMs at various levels for the intact model and all three fixation strategies; (**b**) disc stress at various levels for the intact model and the three fixation strategies, highlighting the balance of junctional stress; (**c**) at the L3–L4 transition segment, the dynamic Dynesys demonstrated reduced ROM, disc stress, and FCF compared with the non-fixated condition. DTO, dynamic-transitional optima; ROM, range of motion; FCF, facet contact force.

The application of DTO fixators, along with one- and two-level static fixators, demonstrated that junctional problems were more common at the superior level (L2–L3) than at the inferior level (L5–S1) (Figure 5a,b). This system outperformed the two-level static fixation in mitigating these issues. When normalized to the fully degenerative non-instrumented model (100%), the hybrid construct demonstrated 30%, 64%, 23%, and 16% higher ROM at the L3–L4 transition segment during flexion, extension, lateral bending, and axial rotation, respectively, than the two-level static fixation. At the L3–L4 transition segment, disc stress in the DTO system increased by 39%, 87%, 28%, and 34% under flexion, extension, lateral bending, and axial rotation, respectively, over the two-level static fixation. Conversely, at the L2–L3 supra-adjacent segment, the two-level static model showed the ROM increased, ranging from 5% (rotation) to 36% (extension), and the disc stress increased from 21% (flexion) to 40% (lateral bending) compared with the DTO model. Compared with one-level static fixation that excluded L3–L4 instrumentation, the DTO construct exhibited 2–37%, 22–89%, and 10–187% reductions in ROM, disc stress, and FCF, respectively, at the transition segment compared with the two-level static fixation (Figure 5c). Overall, the DTO hybrid model restricted the ROM less but induced greater stress on the disc/facet joints at the transition segment compared with the conventional static stabilization.

### 3.2. Mechanical Characterization of DTO Systems in the Load-Sharing Trade-Off Zone Under Varying Disc Degeneration Conditions

**Figure 6:**
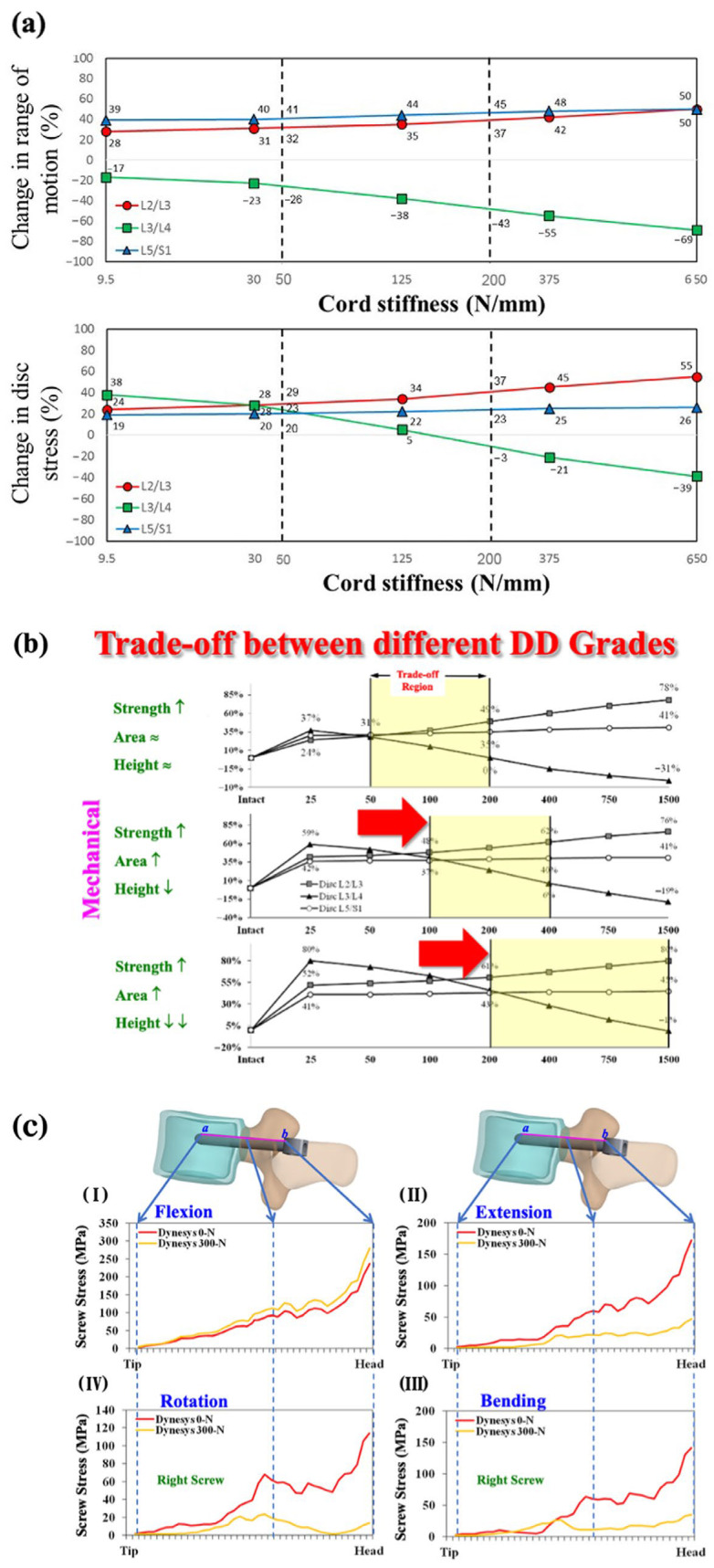
Effect of disc degeneration and cord pretension on disc stress and screw load. (**a**) The trade-off region of Dynesys cord stiffness was set as 50–200 N/mm because the convergent value of disc stress decussated around 50 N/mm and approached zero around 200 N/mm. (**b**) Variations in balanced disc stress relative to fixator stiffness across the adjacent (L2–L3 and L5–S1) and transition (L3–L4) discs under mild, moderate, and severe disc degenerations. (**c**) The screw stress profile at the bone–screw interfaces at the transition level, under two extreme cord pretension conditions, demonstrated a near-linear pedicle motion trajectory, with maximum stress concentrated at the screw hub during (I) flexion, (II) extension, (III) lateral bending, and (IV) rotation.

For the mild L3–L4 degeneration, the Dynesys cord exhibited a trade-off stiffness range of 50–200 N/mm, where disc stress convergence occurred near 50 N/mm and declined to zero at 200 N/mm [20]. In the normalized fully degenerative model without instrumentation, the ROM (26–43%) and disc stress (−3% to 23%) during flexion remained within the trade-off zone at the L3–L4 transition segment, whereas the corresponding values at the baseline cord stiffness of 650 N/mm were 69% and 39%, respectively (Figure 6a). At the L2–L3 cranial adjacent segment, during flexion, the ROM (32–37%) and disc stress (29–37%) increased relative to the normalized model, compared with 50% ROM and 55% disc stress at the baseline stiffness of 650 N/mm. A higher-stiffness cord was better suited for highly degenerated transition discs (Figure 6b) (because the convergent values of disc stress decussated shifting to higher cord-stiffness levels), maintaining high ROMs while minimizing disc and facet stresses, thereby reducing the junctional problems. However, the trade-off effects of the DTO during extension and lateral bending were confined within a 5% deviation, and the effects during rotation were unchanged for a given spacer length [20].

### 3.3. Stress Profiles at Screw–Spacer and Bone–Screw Junctions in Dynamic Stabilization Systems

The performance of the dynamic component in the DTO hybrid device showed the contact sites along the screw–spacer junction curve during motion around the centre of rotation under 300 N cord pretension (Figure 4d) [26]. In contrast, the pretensioned cord displayed a linear pedicle travel path along the bone–screw interface, with maximal stress concentrated at the screw hub near the posterior pedicle orifice during all motion types (Figure 6c). There was minimal variation in the stress distribution within the 0–300 N/mm range of cord pretension during flexion, compared with extension, lateral bending, and axial rotation [20]. The pedicle screw travel in the dynamic Dynesys system induced shear stress at the screw–vertebral interface, which correlated with the degree of degeneration, potentially contributing to screw loosening. This included cases of poor bone or disc quality [30] or implant failure at the screw hub under high anterior column loading during extreme flexion.

### 3.4. DTO Kinematic Behaviour Under Different Disc Degeneration Conditions

The mechanical response of the motion control unit at the transitional level was also evaluated experimentally under a load-controlled mode by computing the averaged IPT and ID values (Table 1). Following the installation of the DTO device, the IPT values were scaled to 1.18 under vertical axial load and to 1.29 under combined axial load and flexion. In lateral bending, the IPTs were adjusted to 0.90 and 0.95 on the stretching and contraction sides, respectively, under combined axial bending loading. There were no statistically significant differences in the IPT values induced by the DTO across varying levels of disc degeneration (all *p* > 0.05). In contrast, borderline significant differences were observed in the ID values under axial load alone or when combined with lateral bending. This finding is consistent with the design effects of the longitudinal cord/spacer, which is intended to minimize the angular deviation [20]. Notably, the IPT axial translation appeared to influence the dynamic performance of the DTO system more than the ID vertical displacement.

### 3.5. Analysis of Clinical Cases with L3–L4–L5 Dynamic Hybrid Fixation

**Figure 7:**
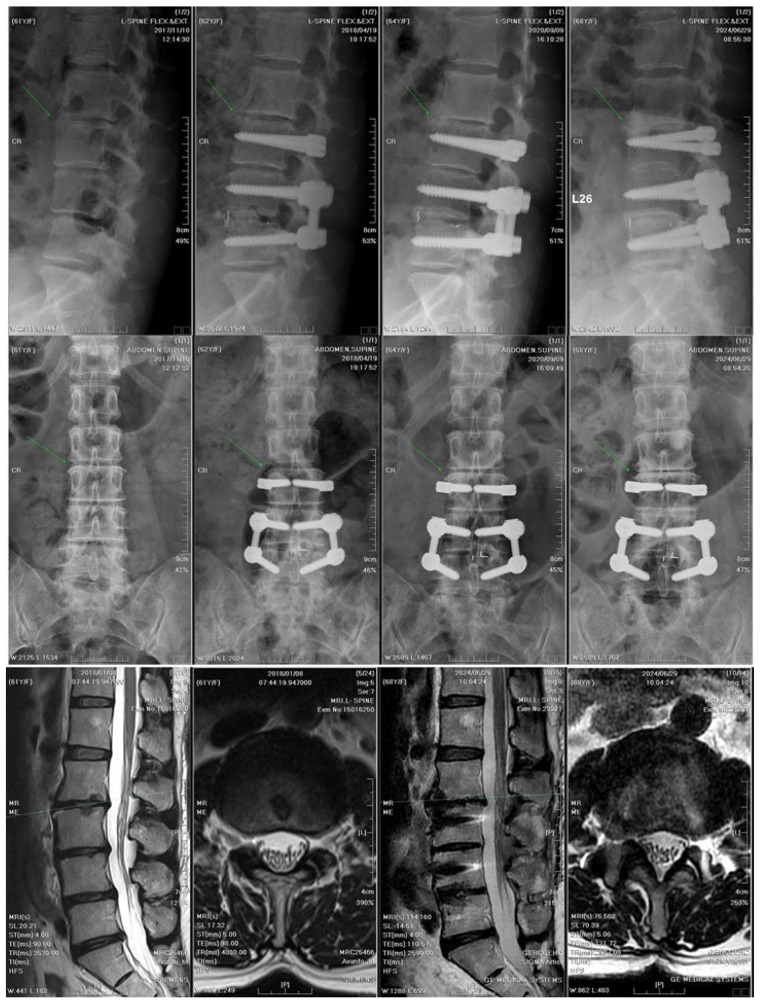
Radiographic and MRI evidence of delayed degeneration with DTO fixation. An illustrative case of a 65-year-old woman who underwent DTO fixation. The upper and middle panels show serial X-rays from left to right (preoperative, 1-month postoperative, 18-month postoperative, and 6-year postoperative), revealing the course of degenerative changes. The L3–L4 transition segment exhibited delayed degeneration compared with the adjacent non-instrumented L2–L3 segment, where osteophytes and disc space narrowing are indicated by green arrows. The bottom panel displays MRI images (sagittal and axial views) of the L3–L4 segment showing only mild degenerative changes over a 6-year follow-up period. The findings indicate that dynamic stabilization slows disc degeneration. DTO, dynamic-transitional optima; MRI, magnetic resonance imaging.

A total of 31 older patients underwent the novel MIS procedures, with no revision surgeries required, except for two asymptomatic cases of screw loosening observed at the final follow-up. The mean posterior disc heights showed a gradual decrease over time; however, the L3–L4 segment exhibited a relatively smaller decline compared with the adjacent segments (−29% [L3–L4] vs. −35% [L2–L3] and −34% [L5–S1]; *p* = 0.549). Regarding the 4-year changes in listhesis distance, only the L3–L4 segment demonstrated a significantly smaller increase compared with the other levels (0.18 cm [L3–L4] vs. 0.27 cm [L2–L3] and 0.20 cm [L5–S1]; *p* = 0.001). Regarding the dynamic angular changes between flexion and extension, a significant reduction from the preoperative values was observed exclusively at the L3–L4 segment at the 4-year follow-up (6.58° ± 3.78° pre-op vs. 4.34° ± 3.29° at final follow-up; *p* = 0.023). Overall, the L3–L4 segment took a longer period to exhibit significant reductions in the posterior disc height, spondylolisthesis, and dynamic angular range relative to the L2–L3 and L5–S1 segments over the 4-year postoperative follow-up (Table 2, Figure 7). These findings suggest that over time, the dynamic component of the DTO hybrid device may contribute to an ASD delay, while preserving the overall lumbar lordosis.

## 4. Discussion

The compliant design of the Dynesys cord–spacer system was developed in multiple configurations to maintain segmental mobility and enhance the load distribution at the transitional level [31]. Within the “trade-off region,” the Dynesys cord stiffness was reduced to investigate the biomechanical effects, which suggested that extreme vertebral loads and flexion could cause material fatigue, potentially leading to implant loosening [19]. Within the trade-off stiffness range (50–200 N/mm), the cord increased segmental motion at L3–L4, while balancing stress at the adjacent L2–L3 and L5–S1 discs, resulting in stabilized values of 17% (43% → 26%) for the ROM and 26% (−3% → 23%) for disc stress during flexion. (Figure 6a). At the L3–L4 transitional level, the trade-off cord–spacer construct enhanced the biomechanical behaviour, with the ROM increasing by 2.7% in rotation and up to 12.7% in flexion, the disc stress decreasing by 4.1% in flexion and 12.9% in extension, and the FCF reducing by 4.9% in rotation and 15.6% in extension (Figure 5c). However, cords with higher stiffness performed better in transitional discs exhibiting severe degeneration (Figure 6b) [10,20].

FE analysis of Dynesys performance has shown that the minimization of the intradiscal pressure and posterior annular protrusion depends on the forces exerted by a flexible implant adapting the cord elasticity and spacer geometry (typically 2 mm higher after distraction) [6,7,8,9]. Liu et al. [32] found that the FCF increased by 35% during extension when the Dynesys cord flexion stiffness was increased from 19.0 to 64.5 Nm/deg, which also resulted in a higher load on the screw during flexion and lateral bending. The spacer length primarily affected the extension and lateral bending by balancing loads between the transition and adjacent segments, with minimal influence on rotation. Reduced pretension within the trade-off range enhanced the segmental motion and load distribution, thereby decreasing the stress during flexion and alleviating the FCF during extension through improved contact dispersion along the tube [26]. Conversely, the initial 300 N pretensioned cord created a firmer screw–spacer coupling during flexion; however, the stress-concentrated interface between the screw and vertebra could potentially lead to device pullout with mid-term use [33] (Figure 4d and Figure 6c).

The Dynesys system’s screw–spacer coupling design functions as a fulcrum, leveraging vertebral loads to distribute the stress more evenly across the spinal segment. The contact force at the Dynesys screw–spacer interface was approximately 33% lower than the total vertebral loading, reflecting the remaining 67% contribution from cord tension, paraspinal muscle activity, and gravitational forces under a 300 N pretension condition [26]. Under extreme flexion, the highly stressed Dynesys screws are more prone to loosening in osteoporotic vertebrae, rather than failing due to device fatigue at the screw–spacer junction. Varying the Dynesys cord pretension produced a nearly linear pedicle displacement pattern, with the maximum stress concentrated around the posterior screw head, although pretension changes had minimal influence during flexion (Figure 6c). Clinically, the cord–spacer configuration of the Dynesys device facilitates consistent screw–spacer contact, reducing the surface deformation in cases of individualized disc or facet degeneration [33,34]. However, the dynamic cord–spacer linkage may not adequately resist excessive shear forces in unstable spondylolisthesis, potentially leading to mechanical fatigue under heavy vertebral loads and extreme flexion [12,35,36].

The DTO design aims to normalize intersegmental motion and delay the progression of instability in transitional segments exhibiting mild symptoms that do not yet require arthrodesis. Despite direct biomechanical correlation with long-term clinical outcomes limits, PDS studies suggest promising results. Postoperative outcomes are generally favourable, with radiographic ASD incidences of 6.5–10%—approximately four times lower than the 30–36% observed with fusion procedures alone [35,36,37,38]. Revision rates of up to 9.4% are reported, primarily due to implant breakage and symptomatic ASD, rather than screw loosening, over long-term follow-up. Although ASD incidence varies—partly reflecting the natural course of spinal degeneration—several factors are associated with increased risk, including older age, poor sagittal balance (e.g., loss of lumbar lordosis, leading to greater pelvic tilt and a pelvic incidence–lumbar lordosis discrepancy > 15°), and multilevel hybrid instrumentation [4,35,38]. Notably, the revision risk triples in cases of multilevel fusion due to the spine’s diminished capacity to accommodate biomechanical alterations, especially when segmental lordosis is significantly reduced (by as much as 3.5°) [39].

One pertinent question that arises regarding the study populations from the literature review—highlighting indications and differences in ASD and revision rates (Table 3, [39,40,41,42,43,44,45,46])—is whether ASD progression differs from the natural course of spinal degeneration following MIS when DTO is utilized, given the lower reported reoperation rates [12,14,21]. In response, we proposed an evolving approach involving minimal decompression with critical preservation of the posterior complex (including the spinous processes, paraspinal muscles, and supraspinous ligaments) under a neutral lumbar alignment. Radiologic failure was defined as a decrease of >3 mm in posterior disc height, an intervertebral angle change at flexion of <5°, or a progression of slippage >3 mm compared with the preoperative flexion–extension lateral radiographs. When preserving the tension band mechanism, MIS enhances resistance against flexion moments—particularly in cases with appropriate sagittal balance—resulting in lower energy expenditure and reduced muscle loading. This holds true even in relatively stable grade I spondylolisthesis, especially when the segmental lordosis is restored to >15° [12,35,47].

The IPT and ID parameters characterize the interpedicular motion between paired pedicle screws across the transitional level. Optoelectronic assessment of Dynesys motion characteristics revealed that elongated spacers reduce disc stress and FCF [10]. We employed an optoelectronic tracking technique, which showed that the IPT trajectory in Dynesys cord–spacer linkage is correlated with the degree of disc degeneration (Table 1) [20]. The IPT is not assessed near the rotational centre but instead reflects a combination of rotational and translational motion across the spinal unit (Figure 4c,d). The longitudinal cord pretension was further assessed by measuring the intersegmental mobility (as modelled in FE analysis) and the experimental measurement of device sensitivity under varying degenerative disc conditions. Discrepancies between the numerical and mechanical test outcomes are attributable to the simplified instrumentation model in FE models or challenges in preserving consistent lordotic alignment of the cord–spacer assembly across repeated experiments. The initial preload was critical in ensuring the implant construct stability and halving the von Mises stress, thereby enabling the quantification of IPT or ID differences across varying degrees of disc degeneration. The load borne by posterior hybrid devices is influenced by the anterior disc’s stiffness characteristics under imposed motion. Aligning FE predictions with experimental measurements of DTO performance allows for enhanced sensitivity and clinical relevance, especially under diverse disc and bone conditions. Furthermore, a modified DTO construct employing MIS decompression and critical preservation of posterior complex at the dynamic segment would reduce implant–bone interfacial stress and mitigate ASD risk across a spectrum of degenerative changes.

## 5. Limitations

This study has several limitations. Within the model, the disc–facet strength was varied concurrently, assuming no lordotic progression or degenerative changes. Regarding the PDS designs, the hyper-elastic property of the cord–spacer was neglected, and the FE analysis used a single spacer length. Additionally, pedicle screw loading may have been underpredicted due to the simplified geometry and the absence of detailed threading in the screw model. Interface instability at the bone–screw and screw–spacer junctions was identified as a primary failure mode in dynamic instrumentation systems; however, complex interfacial slippage was difficult to model due to the computational demands of highly convergent nonlinear simulations. The FE analysis also did not address the scenario of a destabilized spine fixed with hybrid devices, and the loading condition was applied solely using a displacement-controlled method. A load-controlled motion analysis was performed to assess the biomechanical variations at the transitional segment under different disc stiffnesses, mainly in flexion and lateral bending. Furthermore, the synthetic testing model did not include the posterior complex or analysis of the bone–screw interfacial stress, nor did it consider ligament constraint or pelvic parameters. From a clinical standpoint, patient selection and surgical precision are critical, particularly when employing MIS techniques for dynamic-transitional optima (DTO) implantation. Long-term clinical relevance remains to be validated, especially in elderly patients undergoing two-level (as opposed to multilevel) hybrid instrumentation. Notably, this study did not include a prospective comparative analysis to assess outcomes over time.

## 6. Conclusions

Dynamic hybrid devices can protect the transitional level but may alter the motion patterns at neighbouring segments—a trade-off issue associated with the Dynesys system, which correlates with the risk of pullout and fatigue failure. A modified DTO construct, combined with preservation of the posterior complex and minimal decompression adjacent to the fusion level, can significantly stabilize the transition segment and reduce implant–bone interfacial complications in various degenerative conditions.

## Figures and Tables

**Table 1:** Interpedicular travel and displacement of the DTO at the transitional level (scale variation method).

Load Condition	IPT				ID			
	A (mm)	B (mm)	B/A	*p*-Value	C (mm)	D (mm)	D/C	*p*-Value
Axial D	0.17 ± 0.10	0.20 ± 0.07	1.18	0.28	−0.01 ± 0.03	−0.07 ± 0.05	7	<0.001
Axial + flexion	0.14 ± 0.05	0.18 ± 0.10	1.29	0.18	−0.01 ± 0.05	−0.04 ± 0.02	4	0.13
Axial + bending (stretching side)	0.52 ± 0.10	0.47 ± 0.04	0.90	0.21	0.07 ± 0.02	0.11 ± 0.01	1.57	0.01 *
Axial + bending (contraction side)	0.66 ± 0.12	0.63 ± 0.09	0.95	0.36	−0.38 ± 0.04	−0.44 ± 0.01	1.16	0.02 *

Note: Stiffness values of 600 N/mm (A, C) and 1100 N/mm (B, D) represent mild degeneration at L3–L4, while 1600 N/mm corresponds to moderate degeneration at L4–L5 in all models. DTO, dynamic-transitional optima; IPT, interpedicular travel; ID, interpedicular displacement. * *p* < 0.05.

**Table 2:** Preoperative and 1-, 2-, and 4-year postoperative radiographic data.

Location	Preoperative	1-Year Postoperative	2-Year Postoperative	4-Year Postoperative
	Mean ± SD	Mean ± SD	Mean ± SD	Mean ± SD
**L2–L3 segment**				
Posterior disc height (cm)	0.62 ± 0.23	0.55 ± 0.20	0.49 ± 0.18	0.37 ± 0.16
Listhesis distance (cm)	0.24 ± 0.09	0.40 ± 0.10	0.42 ± 0.12	0.51 ± 0.12
Angular motion change (degrees)	5.46 ± 3.67	4.99 ± 3.48	5.21 ± 3.32	5.18 ± 3.22
**L3–L4 segment**				
Posterior disc height (cm)	0.58 ± 0.23	0.53 ± 0.21	0.49 ± 0.19	0.39 ± 0.19
Listhesis distance (cm)	0.25 ± 0.09	0.34 ± 0.09	0.37 ± 0.11	0.43 ± 0.09
Angular motion change (°)	6.58 ± 3.78	4.65 ± 3.64	4.26 ± 3.46	4.34 ± 3.29
**L5–S1 segment**				
Posterior disc height (cm)	0.65 ± 0.26	0.58 ± 0.23	0.52 ± 0.19	0.40 ± 0.18
Listhesis distance (cm)	0.28 ± 0.08	0.34 ± 0.10	0.40 ± 0.12	0.48 ± 0.12
Angular motion change (°)	7.90 ± 3.92	8.02 ± 3.78	8.11 ± 3.58	8.19 ± 3.38

SD, standard deviation.

**Table 3:** Literature review of DTO characteristics, focusing on the indications, screw loosening/breakage, ASD, revision rates, complications, and relevant comments.

Author/Year	N	Age (Years)	Follow-Up (Years)	Indications	Surgical Segments	Screw Loosening/Breakage	ASD * Rates	Revision Rates	Complications	Comments
Maserati et al., 2010 [40]	24	49	0.67	Degenerative lumbar disc disease (candidate for DTO: fusion had symptomatic adjacent-level pathology according to discography)	NA	No, but 1/24 had screw malposition	2 (8.3%) had symptomatic degeneration, 1 (4.2%) at the dynamical segment	1 (4.2%) had screw malposition, 3 (12.5%) had revisions of fusion due to persistent symptoms	2 dural tears, 1 symptomatic screw malposition, and 2 infections; not specific to DTO	High risk of ASD development: age > 50 years at time of surgery and fusion to L1–L3 were significant risk factors.
Baioni et al., 2015 [41]	30	47.8	6.1	1. Lumbar stenosis with instability (13) 2. Degenerative spondylolisthesis Meyerding grade I (6) 3. Degenerative disc disease of one or more adjacent levels (6) 4. Mild lumbar degenerative scoliosis (5)	L1–L5 (multi-segments); varied	No	3 (10%) (2 ASDi + 1 asymptomatic retrolisthesis)	2 (6.7%) for ASDi	No mechanical complications, but 3 (10%) ASD cases due to high PI with insufficient lordosis correction	Hybrid fixation may delay the development of ASD, according to spinopelvic measurement and MRI imaging.
Lee et al., 2015 (DTO+ Nflex vs. fusion) [42]	15 vs. 10	60.7 vs. 63.9	4.1 vs. 4.4	Dynamic: symptomatic degenerative segments without instability Fusion: 1. Degenerative segments with spinal instability 2. Spondylolytic spondylolisthesis, more than grade II 3. Severe disc space narrowing	L2–S1, but select two-segment: 15/108 vs. 10/87 for analysis	2 (13.3%) in Nflex system	Adjacent segment pathology defined as 2 mm decrease in posterior disc height: hybrid—6/15 (40%) fusion—7/10 (70%)	NA	NA	1. Hybrid surgery for two-segment disease can maintain original lumbar motion and delay ASD due to the reduced intradiscal pressure. 2. A hybrid stabilization system can preserve lordosis at the operated segments and subsequently reduce compensatory hyperlordosis at the proximal adjacent segment.
Kashkoush 2016 [43]	66	53	5	1. Primary degenerative disc disease 2. Failed back surgery syndrome	L1–L5 (mono-segment, but varied levels)	1 (1.5%) screw breakage	NA	10 (15.2%) converted to fusion: progressive spinal stenosis, disc herniation, continued lower back pain, pseudoarthrosis, progressive spondylolisthesis, symptomatic cage migration, and broken screw	21 (31.2%) had subsequent spine surgery; only mentioned 3 interbody cage migrations, 2 infections, 1 screw breakage, and 1 pseudoarthrosis; others not explained	Carefully selected patients with critical spinal instability and adjacent-level pathology of lesser severity that require decompression (unclearly described procedure) at dynamic stabilized level in 38 cases (56%).
Fay et al., 2018 [39]	30	61.9	2.9	Multilevel lumbar degeneration with or without spondylolisthesis	L1–L5	2/30 (6.7%)	no	2/30 (6.7%) had symptomatic screw loosening	2 screw loosening cases	1. DTO maintains an ideal and neutral lumbar lordosis 2 years after surgery for two-level or multilevel spondylosis. 2. Dynamic stabilization was indicated for simple herniation with preserved motion, whereas fusion was favoured in cases of hard disc formation, significant instability (≥grade II spondylolisthesis), restricted ROM (<3°), or disc collapse. 3. The protective effect against adjacent segment disease remains uncertain, although bridged levels often showed radiographic signs of disc rehydration.
Herren et al., 2018 (DTO vs. fusion) [44]	14 vs. 15	61.78 vs. 60.92	3.14	1. >30 y/o 2. L2–S1 mono-segment LSS or DDD (Modic 1–3) 3. Spondylolisthesis (Meyerding grade 1)	L2–S1 (mono-segment)	Loosening: DTO: 3 (21.4%) vs. fusion: 1 (6.7%) Breakage: DTO: 0 vs. fusion: 1 (6.7%)	DTO: 4 (28.6%)—3 above and 1 below instrument vs. fusion: 4 (26.7%)—4 above instrument	For ASD: DTO: 2 (14.3%) Fusion: 2 (13.3%)	NA	1. Degenerative affection of the adjacent segment: cranial > caudal. 2. Screw loosening remained the primary concern with dynamic systems. 3. The safety of dynamic hybrid devices is unproven when ASD reduction is the primary goal.
Herren et al., 2018 [45]	55	68.9	2.6	NA	L1–L5	Breakage: 8 (14.6%) Loosening: 28 (52.7%)	ASDi: 10 (18.18%) ASD *: 6 (10.91%)	9% for ASDi	ASD: 10.9%; ASDi: 18.8% (conversion to fusion 9%); Screw loosening: 52%; Screw breakage: 10.9%; Rod breakage: 3.64%	1. The initial positive effect of DTO decreased during long-term follow-up (60 months): screw loosening (52%), old age, osteoporosis, device stiffness. 2. It is not clear how far DTOs reduce the force on adjacent segments biomechanically. 3. The mechanical complications do not lead to poorer clinical outcomes.
Ferraro et al., 2020 (Dynesys vs. DTO) [46]	50 vs. 30	47 vs. 48	7.1	1. Recurrent disc herniation 2. Pfirmann 3–4 DDD (Modic 1–2) 3. Low-grade lumbar stenosis 4. Spondylolisthesis Meyerding 1.	NA	In total: 15/80 (19%): 5 asymptomatic + 2 symptomatic screw lucency; 5 screws malpositioned; 1 screw mobilized with superior endplate sinking; 2 Dynesys failure treated with fusion	N/A	8%	2 early infections, 2 transitory radicular disease cases, 7 screw radiolucency cases, 5 screw malposition cases, 1 sagittal imbalance, 1 screw mobilized with superior endplate sinking, 2 implant failure cases	1. Dynamic stabilization modulates abnormal segmental motion rather than correcting spinal deformity. 2. Asymmetric pedicle screws seem to be less tolerated in dynamic stabilization than in spinal fusion because of wrong motion axis. 3. Posterior hybrid stabilization had no clear protective effect on junctional syndrome. 4. Patient selection, cord pretension, and inadequate spacers are the causes of implant failure that may not withstand physiologic loads. 5. Severe disability induced less segmental lordosis up to 3.5 deg.

N, number of patients; NA, not applicable; ASD *, radiological adjacent segment disorder; ASDi, adjacent segment disease; PI, pelvic incidence; MRI, magnetic resonance imaging.

## Data Availability

The data are available from the corresponding author upon reasonable request and with permission from the Taichung Veterans General Hospital. However, restrictions apply regarding the availability of these data, as they are not publicly available.

## Abbreviations

PDS, pedicle-based dynamic stabilization; DTO, dynamic-transitional optima; ASD, adjacent segment disease; FEA, finite element analysis; MIS, minimally invasive surgery; SS, spinal stenosis; ROM, range of motion; FCF, facet contact force; IPT, interpedicular travel; ID, interpedicular displacement.
